# Strong association of the polymorphisms in *PBEF1* and knee OA risk: a two-stage population-based study in China

**DOI:** 10.1038/srep19094

**Published:** 2016-01-11

**Authors:** Minjie Chu, Jiesheng Rong, Yidan Wang, Lin Zhu, Baifen Xing, Yuchun Tao, Xun Zhuang, Yashuang Zhao, Liying Jiang

**Affiliations:** 1 Department of Epidemiology, School of Public Health, Nantong University, Nantong, Jiangsu Province, P. R. China; 2 Second Department of orthopedic Surgery, The Second Affiliated Hospital of Harbin Medical University, Harbin, Heilongjiang Province, P. R. China; 3 Department of Epidemiology, Public Health College, Harbin Medical University, Harbin, Heilongjiang Province, P. R. China; 4 Hongqi Community Health Service Center, Xiangfang District, Harbin, Heilongjiang Province, P. R. China; 5 Department of Health Education, Public Health College, Harbin Medical University, Harbin, Heilongjiang Province, P. R. China

## Abstract

The association of Pre-B cell colony enhancing factor 1 (*PBEF1*) with obesity, together with its pro-inflammatory properties suggests that *PBEF1* might be another crucial mediator that links inflammation with obesity and primary osteoarthritis (OA). We hypothesized that polymorphisms in *PBEF1* may modify the risk of developing OA. Thus we systematically screened 4 tagging polymorphisms (rs4730153, rs2058540, rs3801267 and rs16872158) in *PBEF1* and evaluated the association between the genetic variants and OA risk in a two-stage case-control study including 196 cases and 442 controls in the first stage and 143 cases and 238 controls in the second stage. In the first stage, two SNPs (rs4730153 and rs16872158) were found to be potentially associated with OA risk (*P* < 0.05), which were further confirmed in the second stage with similar effects. After combining the two stages, we found that rs4730153 was significantly associated with decreased risk of OA in an additive genetic model (*P* < 0.05), while rs16872158 showed increased risk of developing OA (*P* < 0.05). Combined analysis of these 2 SNPs showed a significant allele-dosage association between the number of risk alleles and OA risk (*P*_*trend*_ = 5.25 × 10^−5^). These findings indicate that genetic variants in *PBEF1* gene may modify individual susceptibility to OA in the Chinese population.

Primary osteoarthritis (OA) is the most common form of arthritis worldwide and is a major cause of pain and disability in elderly people^1^,^2^,^3^. Although the high prevalence of OA and substantial public health concern, its etiology is not fully understood. An increasing body of evidence suggests that ageing, genetic predisposition, obesity, inflammation and excessive mechanical loading predispose to OA development^4^,^5^,^6^. Recently, growing evidence implicates numerous candidate genes serve as susceptibility loci for which, such as growth differentiation factor 5(*GDF5*), transforming growth factor beta 1(*TGFß1*), estrogen receptor-α (*ESR-α*), *Smad3*, interleukin-6 (*IL-6*), interleukin-1(*IL-1*), the secreted frizzled-related protein 3 (*FRZB*) and the asporin (*ASPN*)^7^,^8^,^9^.

The health economic burden of OA is increasing commensurate with obesity prevalence and longevity. Obesity, considered as a worldwide public health problem with low grade inflammatory status, is one of the well-documented environmental risk factors of knee OA and considered to be moderately associated with hip OA^10^,^11^,^12^,^13^. It is primarily accepted that excess body weight may lead to cartilage degeneration by increasing the mechanical forces across weight-bearing joints. However, several studies have revealed the association between obesity and OA in non-weight-bearing joints such as those in fingers and wrists^6^,^14^,^15^. The mechanisms linking obesity and OA appear to be more complex and multifactorial than expected^16^. Recently, emerging data have suggested that additional metabolic factors released mainly by white adipose tissue may also be responsible for the high prevalence of OA among obese people.

Adipokines, such as leptin, visfatin and resistin, might be also produced in white tissue and altered levels are also contributes to a wide range of obesity-related health problems^17^,^18^,^19^. Adipokines exhibit prominent role in OA pathophysiology, and serve as biomarkers for monitoring disease development and progression following therapeutic interventions^18^,^20^. Obesity is associated with a state of chronic low-grade inflammation. A strong and direct molecular link between obesity and the pathological process of OA has been established^21^. The association of visfatin with obesity, together with its pro-inflammatory properties suggests that visfatin might be another crucial mediator that links inflammation with obesity and OA.

Pre-B cell colony enhancing factor (*PBEF*) (also termed nicotinamide phosphoribosyl transferase, NAMPT or visfatin) was first identified in 1994 as a protein secreted by activated lymphocytes^22^. Visfatin is a multifaceted molecule with proinflammatory cytokine and enzymatic properties^23^,^24^,^25^. It has since been found to have many other functions relating to cell metabolism, inflammation, and immune modulation^26^. Its functions are mainly pro-inflammatory as it potently induces various pro-inflammatory cytokines such as tumor necrosis factor alpha (*TNF-α*) or interleukin-6 (*IL-6*), which indeed reflect a surrogate marker of inflammation^19^,^27^. Visfatin also participates in osteophyte formation^25^,^28^. Visfatin manifested a catabolic function in cartilage and might have an important role in the pathophysiology of OA^29^. Visfatin serve as another inflammatory mediator which affects bone metabolism, and it could be involved in obesity-associated low grade inflammation state^19^,^30^,^31^. The human *PBEF1* gene is located at 7q22.3 and spans 34.7kb with 11 exons, which has been reported to be a linkage region for phenotypes related to obesity and cardiovascular diseases. Also, the coexistence of obesity and OA has increased remarkably nowadays. Given the relevance of visfatin to the pathophysiology of obesity and obesity associated diseases, we hypothesized that *PBEF1* gene might be a candidate gene influencing OA phenotypes. To the best of our knowledge, the *PBEF1* genetic variants determining susceptibility and predicting outcome to knee OA have not been investigated in Asian population. The objective of this study was to evaluate genetic association of the polymorphism in *PBEF1* with susceptibility to knee OA in a northern Chinese population by means of an association study.

## Materials and Methods

### Study population and study design

A two-stage population-based case-control study was conducted. Patients who had at least one of the primary knee joint OA were categorized into OA group, while those without OA were categorized into control group. Participants were population aged 40 years and over living in communities. All of 196 OA cases and 442 controls were recruited from Harbin and designed as the initial discovery stage (the first stage) in this study. Subsequently, independent 143 patients with OA and 238 controls additionally collected from Heilongjiang, were considered as the subjects of the second stage. A questionnaire was designed to collect information from cases and controls regarding general information, occupations and recreational sports activities, previous knee injuries, family history of OA and other diseases, and clinical manifestations of OA (participants with previous knee injury were excluded). Occupations were categorized two groups: non-managerial and managerial/professional (referent). The non-managerial occupations were (1) Service; (2) Farming, Forestry, and Fishing; (3) Precision Production, Craft, and Repair; (4) Operators, Fabricators, and Laborers. The managerial were (5) Managerial and Professional; (6) Technical, Sales, and Administrative Support^32^. Recreational sports activities were categorized three groups: inactive = no reported activity; less active = one to four times a week; more active = five or more times a week^33^. For each patient, radiographic confirmation was assessed using the Kellgren/Lawrence (K/L) grading system^34^. A subject was characterized as having radiographic OA if either knee joint met the Kellgren-Lawrence Scoring System(graded 0–4, where 0 = none; 1 = possible osteophytes only; 2 = definite osteophytes and possible joint space narrowing; 3 = moderate osteophytes and/or definite joint space narrowing; and 4 = large osteophytes, severe joint space narrowing, and/or bony sclerosis). Radiographs were read by a radiologist who was unaware of the clinical findings. Lateral and anterior-posterior knee radiographs were read by 2 radiologists blindly and independently until a consensus was reached. Suspected cases were recalled after the survey and examined by a specialist to ensure validity. Clinical OA not only had definite signs and symptoms but also had radiographic evidence. Clinical patients required the presence of these radiographic findings plus 1 month of joint pain during the previous 12 months. The healthy control subjects were recruited from the survey that had no signs or symptoms of arthritis or joint diseases. All study population underwent a physical examination. Height was measured to the nearest 0.1 cm using a wall-mounted stadiometer. Body weight was measured with a digital scale to the nearest 0.1 kg. Obese cases were BMI ≥ 28 kg/m^2^ and non-obese cases were BMI ≤ 28 kg/m^2^.

The study was reviewed and approved by the Ethics Committee of Harbin Medical University, and written informed consent was obtained from every subject. The methods were carried out in accordance with the approved guidelines. Secondary OA patients such as inflammatory arthritis, rheumatoid, bone fracture and developmental dysplasia were excluded.

### SNPs selection

Based on the NCBI database(http://www.ncbi.nlm.nih.gov/projects/SNP), public HapMap SNP database(phase II +III Feb 09, on NCBI B36 assembly, dbSNP b126) and the Haploview 4.2 software, common SNPs (Minor allele frequency, MAF ≥ 5% in Chinese Han population) were screened in *PBEF1* gene regions. SNPs with low linkage disequilibrium (LD) analysis (*r*^2^ < 0.8) were retained. As a result, 4 tagging SNPs (rs4730153, rs2058540, rs3801267 and rs16872158) were finally determined to perform genotyping. However, rs2058540 was excluded because of design failure.

### Genotyping

Peripheral blood was collected from each subject only after obtaining signed informed consent, and genomic DNA was extracted from the samples by a DNA extraction Kit (Qiagen, Valencia, CA). Genotyping analysis in the first stage was performed using the iPLEX Sequenom MassARRAY platform (Sequenom, Inc), A TaqMan allelic discrimination assay (Applied Biosystems, Inc.) was used for the second stage. The following series of methods were used to control the quality of genotyping: (i) case and control samples were mixed on each plate; (ii) genotyping was performed blinded to case or control status; (iii) two water controls were used in each plate as blank controls; and (iv) 5% of the samples were randomly selected for repeat genotyping, as blind duplicates, and the reproducibility was 100%.

### Statistical analysis

The *χ*^2^ test for categorical variables and Student’s t-tests for continuous variables were used to analyze distribution differences of demographic characteristics, selected variables and genotypes between cases and controls. Hardy-Weinberg equilibrium (HWE) for the distribution of each SNP was evaluated using the goodness-of-fit *χ*^2^ test by comparing the observed genotype frequencies with the expected ones. Odd ratios (ORs) and their 95% confidence intervals (CI) were calculated by using logistic regression analyses to evaluate the association between SNPs and OA risk with an adjustment for age, gender, BMI, occupation and physical activity. To examine the differences between subgroups, the *χ*^2^- based Q-test was used to test the heterogeneity of effect sizes (ORs and 95% CIs) derived from corresponding subgroups. All of the statistical analyses were performed with Stata Version 10.0 software (Stata, College Station, TX).

## Results

The distribution of selected characteristics between OA cases and controls was summarized in Table 1. In Brief, the gender between cases and controls were comparable in each stage (*P* > 0.05). The mean age of cases was higher than that of controls in each stage. Compared with controls, OA cases had a higher rate of overweight (39.29% *vs* 34.84% in the first stage; 37.76% *vs* 31.93% in the second stage) and obesity (26.02% *vs* 18.78% in the first stage; 24.48% *vs* 15.13% in the second stage). In addition, the smoking status, occupation and physical activity between cases and controls were comparable in each stage (*P* > 0.05).

The basic information of the 3 SNPs were shown in Supplementary Table 1, the success rates of genotyping for these polymorphisms were all above 98%. The observed genotype frequencies for these SNPs were all in agreement with HWE (*P* > 0.05) (Table 2). As shown in Table 2, in the first stage, among the 3 SNPs, logistic regression analyses revealed that the variant alleles of rs4730153 (*P* = 0.016 with adjustment for age, gender, occupation and physical activity; *P* = 0.042 with adjustment for BMI, gender, occupation and physical activity) and rs16872158 (*P* = 0.028 with adjustment for age, gender, occupation and physical activity; *P* = 0.013 with adjustment for BMI, gender, occupation and physical activity) were significantly associated with OA risk in additive genetic model, and showed a suggestive association with the susceptibility to OA. Therefore, we further tested the association of these two SNPs with OA risk in the second stage case–control study comprising independent 143 OA cases and 238 controls. As shown in Table 2, both SNPs showed consistent associations with OA in the second stage samples (rs4730153: *P* = 0.048 with adjustment for age, gender, occupation and physical activity; rs16872158: *P* = 0.020 with adjustment for BMI, gender, occupation and physical activity) as that observed in the first stage samples. To increase the statistic power, we combined the two stages and found that minor allele (A) of rs4730153 was significantly associated with a decreased risk of OA in additive genetic model (*P* = 0.002 with adjustment for age, gender, occupation and physical activity; *P* = 0.013 with adjustment for BMI, gender, occupation and physical activity), and minor allele (A) of rs16872158 was significantly associated with an increased risk of OA in additive genetic model (*P* = 0.004 with adjustment for age, gender, occupation and physical activity; *P* = 0.001 with adjustment for BMI, gender, occupation and physical activity). The associations of rs4730153 and rs16872158 and OA risk were still significant after correction for multiple testing (n = 3) with *P* values less than 0.017. However, there were no obvious evidences of significant association between the remaining SNP rs3801267 and OA risk.

In our present study, rs4730153 and rs3801267 were found to be in high LD (*r*^*2*^ = 0.797) (Supplementary Table 2). We then conducted conditional regression analysis and found that, after controlling rs3801267, the effect of rs4730153 on OA risk remained significant (*P* < 0.05), suggesting that the effects of the two SNPs were not highly correlated.

Furthermore, in the stratification analysis, the association between rs4730153, rs16872158 and OA risk were evaluated in subgroups based on age and BMI. As shown in Supplementary Table 3, a significant difference between subgroups of BMI was observed for the association of rs16872158 with OA risk (*P* for heterogeneity = 0.032).

Notably, considering the significant differences in distribution of age and BMI between case and control groups, we further investigated whether the effect of the identified variants on OA was modified by age and BMI. Interaction analyses showed that rs16872158 interacted multiplicatively with age to contribute to OA risk (interaction *P* = 3.32 × 10^−9^) (Supplementary Table 4A). Similar effect was observed for rs16872158 and BMI that interacted multiplicatively to contribute to OA risk (interaction *P* = 1.81 × 10^−4^) (Supplementary Table 4B). However, no significant interaction was observed for another SNP rs4730153 and age on OA risk, neither nor rs4730153 and BMI.

In view of the modest or small effect of each individual locus, we further conducted a combined analysis to evaluate the cumulative effect of the 2 identified tagging SNPs with the combination of the two stages. We found that the more unfavorable alleles the subjects carried, the higher risk of developing OA they have, suggesting a allele-dosage effect (*P*_trend_ = 5.25 × 10^−5^; Table 3). Individuals carrying ‘2’ unfavorable alleles had a significantly 80% increased risk of developing OA (OR = 1.80, 95% CI: 1.18–2.74) compared with those having ‘0–1’ unfavorable allele, while the elevated risk was more evident among subjects having ‘3–4’ unfavorable alleles (OR = 3.22, 95% CI: 1.83–5.69). Besides, since using the most common group as a reference would make the results more meaningful, we also calculated joint effect of the 2 SNPs using ‘2’ unfavorable alleles as a reference group and found that, as expected, those with ‘0–1’ risk allele had a decreased risk (OR = 0.56, 95% CI: 0.36–0.85) while those with ‘3–4’ unfavorable alleles showed higher risk (OR = 1.79, 95% CI: 1.15–2.80).

## Discussion

In our present study, we evaluated the association of 3 tagging polymorphisms in the *PBEF1* with OA risk in a two-stage case-control study in combined data sets of 339 OA cases and 680 controls. Two SNPs (rs4730153 and rs16872158) were identified to be significant associated with knee OA risk. To the best of our knowledge, this is the first association study of polymorphisms in *PBEF1* and OA risk in Asian.

Visfatin is a novel adipokine that is produced by the adipose tissue, which simultaneously facilitates adipogenesis and has insulin-mimetic properties. Epidemiological studies suggest that plasma level of visfatin is increased in parallel with obesity and it might be useful as a surrogate marker of pro-inflammatory state. It might be good news for obesity-related disease treatment, including metabolic syndrome, type 2 diabetes mellitus and OA.

In addition, we also performed functional annotation for the 2 marker SNPs, as well as those tagged by the 2 marker SNPs (*r*^2^ > 0.8) based on DNase-seq database and RegulomeDB (http://regulome.stanford.edu/) (Supplementary Table 5). Among the 2 marker SNPs and those 45 SNPs highly correlated with which, 11 SNPs are within open chromatin regions associated with gene regulatory elements. Furthermore, 18 SNPs located in motifs may influence the binding of specified transcription factors. These suggest that the 2 marker SNPs and those tagged by which may transcriptionally modulate the expression of *PBEF1* gene. It is plausible that variants in the 2 SNPs or SNPs in high LD with which, collaboratively result in the aberrant activities of certain transcription factors. In turn, these factors interactively regulate the expression of the *PBEF1* gene, hence regulating OA development through different mechanisms. However, no experimental evidence validates this hypothesis and future functional studies are warranted to clarify this point.

There are several strengths in the present study. First, by exerting much effort to identify knee OA related variants in *PBEF1*, a well-designed two-stage population-based study was performed, and two SNPs (rs4730153 and rs16872158) were successfully replicated in the second stage, leading to the combined *P* values of which all significant after correction for multiple testing. Second, OA phenotypic definitions, which reflect a different subset of OA, have been shown to influence the ability to detect genetic associations. The focus on radiographic features and clinical diagnosis in the definition is an advantage because it is more stringent and clinically relevant in this study.

However, several limitations of our study also need to be addressed. First, one SNP rs2058540 was excluded because of design failure, which may limit the success rates of genotyping. Second, exact biological mechanism of the promising variants could not be annotated and the real causal variant was unclear. Third, OA is a multifactorial disease with strong genetic component with various heritability estimates depending on different joint sites. We only evaluates the risk of knee OA and *PBEF1* gene polymorphisms because of failure to X-rays examining in other joint sites, and the results can not be well generalized to other joints. Nevertheless, there is reason to believe that the findings are of considerable credibility and veracity. Fourth, other known genetic risk factors for OA haven’t been assessed and adjusted for in our present study. Additionally, in the current sample size (339 OA cases and 680 control subjects), the statistical power is about 53% when detecting an effect size of 1.50 with α-level of 0.05 for the association of the identified SNPs with OA risk. Further studies with larger sample sizes are needed to evaluate our findings, especially in non-Chinese populations.

In summary, our study investigated the role of genetic variants in *PBEF1* gene in OA development in the Chinese population. Given the fact that genetic factors may vary with different disease pattern, severity, gender and populations, the results for the studied SNPs need further larger scale replication study in other populations. Our results suggested for the first time, that rs4730153 and rs16872158 may modify the risk of OA development. Further studies are warranted to validate and extend our findings, and to re-sequence the identified regions and to evaluate the potential functional significance of the loci. Moreover, further studies should focus on sex-specific mechanisms on the etiology of knee OA development and elucidate whether *PBEF1* gene can be targeted for prevention and therapy strategies to mitigate the obviously increased prevalence of knee OA.

## Additional Information

**How to cite this article**: Chu, M. *et al.* Strong association of the polymorphisms in *PBEF1* and knee OA risk: a two-stage population-based study in China. *Sci. Rep.* **6**, 19094; doi: 10.1038/srep19094 (2016).

## Supplementary Material

Supplementary Information

## Figures and Tables

**Table 1:** Distributions of select variables in OA cases and controls.

Variables	The first stage			The second stage		
	Cases	Controls	*P* Value	Cases	Controls	*P* Value
	(n = 196) N (%)	(n = 442) N (%)		(n = 143) N (%)	(n = 238) N (%)	
Age, year (mean ± SD)	62.19 ± 8.76	57.17 ± 9.19	<0.001	62.10 ± 9.00	56.95 ± 9.07	<0.001
<57^a^	56 (28.57)	213 (48.19)	<0.001	40 (27.97)	115 (48.32)	<0.001
≥57^a^	140 (71.43)	229 (51.81)		103 (72.03)	123 (51.68)	
Gender						
Male	48 (24.49)	139 (31.45)	0.090	31 (21.68)	65 (27.31)	0.273
Female	148 (75.51)	303 (68.55)		112 (78.32)	173 (72.69)	
BMI						
<24	68 (34.69)	205 (46.38)	0.015	54 (37.76)	126 (52.94)	0.009
24 ≤ BMI < 28	77 (39.29)	154 (34.84)		54 (37.76)	76 (31.93)	
≥28	51 (26.02)	83 (18.78)		35 (24.48)	36 (15.13)	
Smoking status						
Ever	46 (23.47)	119 (27.23)	0.330	31 (21.68)	57 (24.26)	0.617
Never	150 (76.53)	318 (72.77)		112 (78.32)	178 (75.74)	
Drinking status						
Ever	52 (27.81)	134 (32.60)	0.254	41 (28.67)	94 (39.50)	0.036
Never	135 (72.19)	277 (67.40)		102 (71.33)	144 (60.50)	
Occupation^b^						
Managerial	85 (44.50)	218 (49.77)	0.224	57 (39.86)	116 (48.74)	0.092
Non-managerial	106 (55.50)	220 (50.22)		86 (60.14)	122 (51.26)	
Physical activity^c^						
Inactive	68 (34.69)	177 (40.04)	0.090	54 (37.76)	95 (39.92)	0.583
Less active	78 (39.80)	185 (41.86)		46 (32.17)	83 (34.87)	
More active	50 (25.51)	80 (18.10)		43 (30.07)	60 (25.21)	

^a^Median age in control group.

^b^The non-managerial occupations: Service; Farming, Forestry, and Fishing; Precision Production, Craft, and Repair; Operators, Fabricators, and Laborers. The managerial occupations: Managerial and Professional; Technical, Sales, and Administrative Support.

^c^Inactive = no reported activity; Less active = one to four times a week; More active = five or more times a week.

**Table 2:** Associations between 3 tagging SNPs in *PBEF1* gene with OA risk.

SNP	Allele^a^	Study	Cases^b^	Controls^b^	HWE^c^ (case/control)	OR_add_ (95% CI)^d^	*P*_add_^d^	OR_add_ (95% CI)^e^	*P*_add_^e^
rs4730153	G/A	The first stage	170/25/0	350/85/4	0.34/0.64	0.55 (0.34–0.90)	0.016	0.61 (0.38–0.98)	0.042
		The second stage	124/16/1	189/43/3	0.55/0.76	0.55 (0.31–0.99)	0.048	0.67 (0.38–1.19)	0.173
		Combined				0.55 (0.38–0.80)	0.002	0.63 (0.44–0.91)	0.013
rs16872158	T/A	The first stage	164/30/1	400/39/2	0.77/0.33	1.73 (1.06–2.82)	0.028	1.84 (1.14–2.97)	0.013
		The second stage	119/21/1	217/18/1	0.94/0.36	1.86 (0.98–3.51)	0.056	2.11 (1.13–3.95)	0.020
		Combined				1.77 (1.20–2.60)	0.004	1.92 (1.32–2.80)	0.001
rs3801267	T/A	The first stage	169/25/0	362/71/5	0.34/0.47	0.66 (0.41–1.08)	0.098	0.74 (0.46–1.18)	0.205

^a^Major allele/Minor allele.

^b^Wild-type homozygote/heterozygote/variant homozygote.

^c^Hardy–Weinberg equilibrium test.

^d^Logistic regression with adjustment for age, gender, occupation and physical activity in additive model.

^e^Logistic regression with adjustment for BMI, gender, occupation and physical activity in additive model.

**Table 3:** Joint effect of rs4730153 and rs16872158 on OA risk.

Number of risk allele^a^	Case (n = 334) n (%)	Control (n = 671) n (%)	Adjusted OR (95% CI)^b^	*P*^b^
0–1	36 (10.78)	127 (18.93)	Reference	–
2	251 (75.15)	492 (73.32)	1.80 (1.18–2.74)	6.30 × 10^−3^
3–4	47 (14.07)	52 (7.75)	3.22 (1.83–5.69)	5.45 × 10^−5^
Trend test				5.25 × 10^−5^
0–1	36 (10.78)	127 (18.93)	0.56 (0.36–0.85)	6.30 × 10^−3^
2	251 (75.15)	492 (73.32)	Reference	–
3–4	47 (14.07)	52 (7.75)	1.79 (1.15–2.80)	1.06 × 10^−2^

^a^rs4730153-G and rs16872158-A alleles were assumed as risk alleles.

^b^The reference group was those with ‘0–1’or ‘2’ risk alleles. Adjusted for age, gender, BMI, occupation and physical activity.
