# Supplementary material for: Strong association of the polymorphisms in PBEF1 and knee OA risk: a two-stage population-based study in China
Source: Sci Rep. 2016 Jan 11;6:19094. doi: 10.1038/srep19094 (PMC4707545; doi:10.1038/srep19094)
Supplement: Supplementary Information [file srep19094-s1.pdf]

Strong association of the polymorphisms in *PBEF1* and knee OA risk: a two-stage population-based study in northern China

Minjie Chu[1], Jiesheng Rong[2], Yidan Wang[1], Lin Zhu[3], Baifen Xing[4], Yuchun Tao[5], Xun Zhuang[1], Yashuang Zhao[3], Liying Jiang[1, 3]

[1]Department of Epidemiology, School of Public Health, Nantong University, Nantong, Jiangsu Province, P. R. China

[2]Second Department of Surgery, The Second Affiliated Hospital of Harbin Medical University, Harbin, Heilongjiang Province, P. R. China

[3]Department of Epidemiology, Public Health College, Harbin Medical University, Harbin, Heilongjiang Province, P. R. China

[4]Hongqi Community Health Service Center, Xiangfang District, Harbin, Heilongjiang Province, P. R. China

[5]Department of Health Education, Public Health College, Harbin Medical University, Harbin, Heilongjiang Province, P. R. China

Corresponding to: Liying Jiang, Department of Epidemiology, School of Public Health, Nantong University, Nantong, Jiangsu Province, P. R. China
J_meili@126.com
Yashuang Zhao, Department of Epidemiology, Public Health College, Harbin Medical University, Harbin, Heilongjiang Province, P. R. China
zhao_yashuang@263.net

Minjie Chu, Jiesheng Rong and Yidan Wang contributed equally to the paper
Liying Jiang and Yashuang Zhao contributed equally to the paper

Supplementary Table 1. Summary of 3 tagging SNPs in *PBEF1* gene

Gene	SNP	Base change [a]	Location	Genotyping Rate (%)	MAF[b] (case/control)
PBEF1	rs4730153	G>A	intron	99.12	0.06/0.10
	rs16872158	T>A	intron	99.41	0.08/0.05
	rs3801267	T>A	intron	98.92	0.06/0.09

[a] Major allele > Minor allele;

[b] Minor allele frequency;

Supplementary Table 2. Conditional regression analysis and LD values between the 3 SNPs

Primary SNP	Unadjusted *P*[a]	Unadjusted *P*[b]	Adjusted for SNPs	Adjusted *P*[c]	Adjusted *P*[d]	r^2
rs4730153	0.016	0.042	rs16872158	0.024	0.057	0.004
			rs3801267	0.046	0.039	0.797
rs16872158	0.028	0.013	rs4730153	0.041	0.018	0.004
			rs3801267	0.035	0.015	0.003
rs3801267	0.098	0.205	rs4730153	0.188	0.140	0.797
			rs16872158	0.128	0.259	0.003

[a] *P*-value calculated in additive genetic model by logistic regression with adjustment for age, gender, occupation and physical activity

[b] *P*-value calculated in additive genetic model by logistic regression with adjustment for BMI, gender, occupation and physical activity

[c] *P*-value for the primary SNP calculated in additive genetic model by logistic regression with adjustment for age, gender, occupation, physical activity and one additional SNP in column 'Adjusted for SNPs'

[d] *P*-value for the primary SNP calculated in additive genetic model by logistic regression with adjustment for BMI, gender, occupation, physical activity and one additional SNP in column 'Adjusted for SNPs'

Supplementary Table 3. Stratified analysis on the associations of rs4730153 and rs16872158 in *PBEF1* with OA risk.

Characteristics	rs4730153				P_{het} [c]	rs16872158				P_{het} [c]
	Case [a]	Control [a]	OR(95%CI) [b]	P [b]		Case [a]	Control [a]	OR(95%CI) [b]	P [b]	
Age										
<57	80/13/0	260/63/3	0.78(0.41-1.50)	0.458	0.487	81/12/0	294/31/1	1.33(0.65-2.73)	0.439	0.241
≥57	214/28/1	279/65/4	0.59(0.38-0.93)	0.022		202/39/2	323/26/2	2.23(1.38-3.61)	0.001	
BMI										
<24	104/16/1	254/70/3	0.59(0.33-1.03)	0.062	0.207	103/16/2	294/36/1	1.48(0.84-2.62)	0.176	0.032
24≤BMI<28	110/19/0	189/35/4	0.77(0.42-1.41)	0.401		109/20/0	205/20/2	1.33(0.70-2.53)	0.385	
≥28	80/6/0	96/23/0	0.27(0.10-0.72)	0.008		71/15/0	118/1/0	22.93(2.95-178.38)	0.003	

[a] Wild-type homozygote/heterozygote/variant homozygote;

[b] Adjusted for age, gender, BMI, occupation and physical activity where appropriate in additive model;

[c] *P* for heterogeneity

Supplementary Table 4A. The interaction between rs16872158 genotypes and age on OA risk

Age	rs16872158			OR(95%CI)	P [a]
	Genotype	Case	Control		
<57	AA	81	294	1	
<57	CA/CC	12	32	1.40(0.68-2.88)	0.354
≥57	AA	202	323	2.22(1.63-3.02)	3.82×10^{-7}
≥57	CA/CC	41	28	5.60(3.24-9.68)	7.05×10^{-10}
	P for multiplicative interaction				3.32×10^{-9}

[a] *P* value of interaction analysis between rs16872158 and age on OA risk with adjustment for gender, BMI, occupation and physical activity

Supplementary Table 4B. The interaction between rs16872158 genotypes and BMI on OA risk

BMI	rs16872158			OR(95%CI)	P [a]
	Genotype	Case	Control		
<24	AA	103	294	1	
<24	CA/CC	18	37	1.39(0.74-2.61)	0.300
≥24	AA	180	323	1.58(1.17-2.13)	2.80×10^{-3}
≥24	CA/CC	35	23	4.10(2.27-7.41)	2.77×10^{-6}
	P for multiplicative interaction				1.81×10^{-4}

[a] *P* value of interaction analysis between rs16872158 and BMI on OA risk with adjustment for age, gender, occupation and physical activity

Supplementary Table 5. Functional annotation for the 2 marker SNPs and those with strong linkage disequilibrium with the marker SNPs

Number	SNP	marker SNP	r^2	Open chromatin	Regulome DB Score [a]	protein binding
1	rs4730153	rs4730153	1.00		4	POLR2A
2	rs2302559	rs4730153	1.00		5	POLR2A
3	rs10447822	rs4730153	1.00		5	POLR2A
4	rs2041681	rs4730153	1.00		7	
5	rs10808150	rs4730153	1.00		2b	POLR2A
6	rs711438	rs4730153	1.00		5	
7	rs10275206	rs4730153	1.00	Open chromatin	4	BACH1
8	rs3801268	rs4730153	1.00		3a	POLR2A
9	rs3801269	rs4730153	1.00	Open chromatin	4	POLR2A
10	rs10224373	rs4730153	1.00		5	
11	rs3801270	rs4730153	1.00		4	EP300, EBF1
12	rs10953502	rs4730153	1.00		6	
13	rs7807469	rs4730153	1.00		6	
14	rs9034	rs4730153	1.00		4	POLR2A
15	rs10953501	rs4730153	1.00	Open chromatin	3a	CEBPB
16	rs10487820	rs4730153	1.00		5	
17	rs2891846	rs4730153	1.00		5	
18	rs4730152	rs4730153	1.00		5	
19	rs4236582	rs4730153	1.00		7	
20	rs887881	rs4730153	1.00		6	
21	rs12536126	rs4730153	1.00		6	
22	rs7776987	rs4730153	1.00	Open chromatin	4	FOXA1, EP300, NR3C1, MYC
23	rs2016555	rs4730153	1.00		7	
24	rs1013734	rs4730153	1.00		7	

25	rs4382396	rs4730153	1.00		7	
26	rs12534393	rs4730153	1.00		4	MEF2A
27	rs12534383	rs4730153	0.93		3a	MEF2A
28	rs11764463	rs4730153	0.93	Open chromatin	5	
29	rs3801272	rs4730153	0.93		6	
30	rs2058539	rs4730153	0.93		6	
31	rs3801267	rs4730153	0.93		5	
32	rs3779204	rs4730153	0.93	Open chromatin	5	
33	rs4730155	rs4730153	0.93	Open chromatin	4	CEBPB, POLR2A, SMARCA4
34	rs3801266	rs4730153	0.93	Open chromatin	4	CEBPB, POLR2A, SMARCA4, TBP, SAP30, CHD1, MAX, HDAC1
35	rs17373587	rs4730153	0.93		6	
36	rs2160065	rs4730153	0.87		6	
37	rs4451226	rs4730153	0.87	Open chromatin	5	
38	rs3823744	rs4730153	0.86	Open chromatin	4	
39	rs10953503	rs4730153	0.86		4	EP300, EBF1
40	rs12674432	rs4730153	0.86		5	
41	rs10261830	rs4730153	0.86		7	
42	rs2110385	rs4730153	0.86		7	
43	rs35533454	rs4730153	0.86		2c	NR3C1
44	rs28605264	rs4730153	0.86	Open chromatin	5	
45	rs10240418	rs4730153	0.86		4	
46	rs6944744	rs4730153	0.86		6	
47	rs16872158	rs16872158	1.00		5	POLR2A

[a] Description of Regulome DB :2b, TF binding + any motif +DNase footprint + DNase peak; 2c, TF binding + matched motif + DNase peak; 3a, TF binding + any motif + DNase peak; 4, TF binding + DNase peak; 5, TF binding or DNase peak; 6, Motif hit; 7, No data supporting
